# Burnout as a Predictor of Job Satisfaction in Peruvian Nurses: The Mediating Role of Work Engagement

**DOI:** 10.3390/nursrep16020063

**Published:** 2026-02-13

**Authors:** Irene J. Escalante-Zúñiga, Elizabeth Pérez-Flores, María Teresa Cabanillas-Chávez, Liset Z. Sairitupa-Sanchez, Sandra B. Morales-García, Oriana Rivera-Lozada, Wilter C. Morales-García

**Affiliations:** 1 Unidad de Salud, Escuela de Posgrado, Universidad Peruana Unión, Lima 15102, Peru; 2 Unidad de Psicología, Escuela de Posgrado, Universidad Peruana Unión, Lima 15102, Peru; 3 Medicina Humana, Universidad Señor de Sipán, Chiclayo 14001, Peru; 4 Vicerrectorado de Investigación, Universidad Señor de Sipán, Chiclayo 14001, Peru; riveraoriana@uss.edu.pe; 5 Dirección General de Investigación, Universidad Peruana Unión, Lima 15102, Peru; 6 Facultad de Teología, Universidad Peruana Unión, Lima 15102, Peru

**Keywords:** burnout, work engagement, job satisfaction, nurses, JD-R model

## Abstract

**Background**: Burnout and job satisfaction are widely studied phenomena within the field of occupational health, particularly among nursing professionals exposed to high work demands. Work engagement has been identified as a potential mediator that may buffer the negative effects of burnout on job satisfaction. However, in the Peruvian context, empirical evidence on this relational dynamic remains limited. **Objective**: The objective of this study is to examine the mediating role of work engagement in the relationship between burnout and job satisfaction among Peruvian nurses using a structural equation modeling (SEM) approach. **Methods**: An explanatory study was conducted with a sample of 230 Peruvian nurses (M = 41.22, SD = 11.65). Data were analyzed using structural equation modeling. **Results**: Burnout showed significant negative correlations with work engagement (r = −0.47, *p* < 0.01) and job satisfaction (r = −0.41, *p* < 0.01), while work engagement was positively associated with job satisfaction (r = 0.79, *p* < 0.01). The structural model demonstrated a good fit (CFI = 0.96, TLI = 0.95, RMSEA = 0.06, and SRMR = 0.04). The model also indicated solid overall fit and revealed a significant indirect effect of burnout on job satisfaction through engagement, accounting for approximately 24% of the variance in engagement and 80% of the variance in job satisfaction. **Conclusions**: The findings confirm that work engagement fully mediates the relationship between burnout and job satisfaction among Peruvian nurses, serving as a key protective psychosocial resource. These results reinforce the Job Demands–Resources (JD-R) model and highlight the importance of implementing organizational interventions aimed at strengthening work engagement as a strategy to improve satisfaction and well-being in demanding healthcare settings.

## 1. Introduction

Burnout and work engagement among nurses are topics of growing interest in occupational health research, particularly due to the intense demands and inherent stress of the profession. Burnout, or occupational burnout syndrome, is characterized by emotional exhaustion, depersonalization, and a reduced sense of personal accomplishment, and it emerges when job demands chronically exceed the individual’s available resources [1]. In contrast, work engagement is defined as a positive, persistent affective–cognitive state characterized by vigor, dedication, and absorption in one’s work [2]. The work environment of nurses—marked by long shifts, heavy workloads, and continuous exposure to emotionally demanding situations—is consistently associated with elevated levels of burnout and with variations in levels of work engagement [3,4]. During the COVID-19 pandemic, these factors intensified markedly. Several studies have documented substantial increases in emotional exhaustion, depersonalization, and turnover intention among nurses exposed to high care loads, resource shortages, and sustained infection risks [5,6,7]. At the same time, work engagement has been observed to function as a psychological resource that buffers the negative impact of job demands on health and well-being, insofar as it fosters positive emotions, a sense of purpose, and resilience in the face of stress [8,9].

The relationship between burnout and job satisfaction has been described as complex and multidimensional. In general, high levels of burnout are linked to lower job satisfaction, poorer organizational climate, increased absenteeism, and higher turnover intention [10,11,12]. From a classical perspective, job satisfaction is understood as a global or specific evaluation that employees make of their job and work context, integrating factors such as working conditions, interpersonal relationships, and rewards, which directly influence their motivation and their decision to remain in the organization [13]. Within this framework, different studies have shown that organizational resources such as structural empowerment, perceived fairness, supervisor support, and development opportunities are associated with lower burnout and higher job satisfaction [14,15,16].

Work engagement, in turn, has been shown to be closely and positively related to job satisfaction in various care settings. Nurses with higher levels of vigor, dedication, and absorption generally report greater satisfaction with their work and a lower likelihood of leaving the organization [17,18]. Studies in hospital and critical care services indicate that even in high-demand contexts, work engagement is associated with better indicators of well-being and lower professional burnout [12,19]. However, there are also reports of scenarios in which the relationship between engagement and satisfaction is not statistically significant, suggesting the influence of specific contextual factors such as the nature of the service or organizational policies [20,21].

In the Peruvian healthcare context, the relationship between burnout syndrome, work engagement, and job satisfaction among nurses presents a complex and multifaceted picture. Recent studies have shown that burnout and other job stressors negatively affect subjective well-being and perceived job satisfaction, whereas personal resources such as professional self-efficacy and organizational resources such as institutional support foster higher levels of engagement and better performance [22,23]. Likewise, research conducted with Peruvian nurses has shown that, despite limitations in administrative policies and benefits, personal development and task performance can constitute important sources of satisfaction; in fact, higher levels of life satisfaction have been found among substitute nurses compared to permanent staff, highlighting the role of interpersonal relationships and opportunities for professional growth [24]. Furthermore, it has been noted that in emergency and prehospital care services, levels of burnout do not always translate directly into lower job satisfaction, suggesting the presence of specific resources—such as sense of purpose or team support—that modulate this relationship in the Peruvian context [20]. Taken together, these national empirical findings indicate that Peruvian nurses constitute a group particularly exposed to high job demands, while also underscoring the central role of personal and organizational resources in shaping their work engagement and job satisfaction. However, despite these advances, empirical evidence that jointly examines burnout, work engagement, and job satisfaction within an explanatory mediation model among Peruvian nurses remains limited, especially when theoretical frameworks such as the Job Demands–Resources Model are considered. From a theoretical standpoint, the Job Demands–Resources (JD-R) Model provides a relevant framework for understanding these relationships. This model proposes that job demands (e.g., work overload, time pressure, emotional demands) tend to activate a strain process that leads to burnout, whereas job and personal resources (such as social support, autonomy, self-efficacy, or transformational leadership) promote a motivational process that enhances work engagement and produces positive outcomes, including higher performance and job satisfaction [25,26]. Consequently, burnout can be understood as the manifestation of a sustained imbalance between demands and resources, whereas work engagement reflects the capacity to appropriately mobilize available resources to maintain energy, involvement, and concentration in work tasks [27].

Applied to the nursing context, the JD-R model suggests that the high care demands inherent in hospital settings increase the risk of burnout, but that the presence of adequate resources (such as organizational support, professional recognition, and development opportunities) can strengthen work engagement and, through it, enhance job satisfaction [3,4]. From this perspective, work engagement is not only conceived as a desirable outcome but also as a psychological mechanism that mediates the relationship between environmental stressors (burnout) and positive work outcomes (job satisfaction), by transforming the work experience into one that is more meaningful and energizing [2,27]. In this way, the JD-R model offers a robust conceptual framework that allows integration of international and Peruvian evidence, articulating how the demands inherent to nursing work and the resources available in the national healthcare context converge to produce differing levels of burnout, engagement, and job satisfaction.

**Figure 1:**
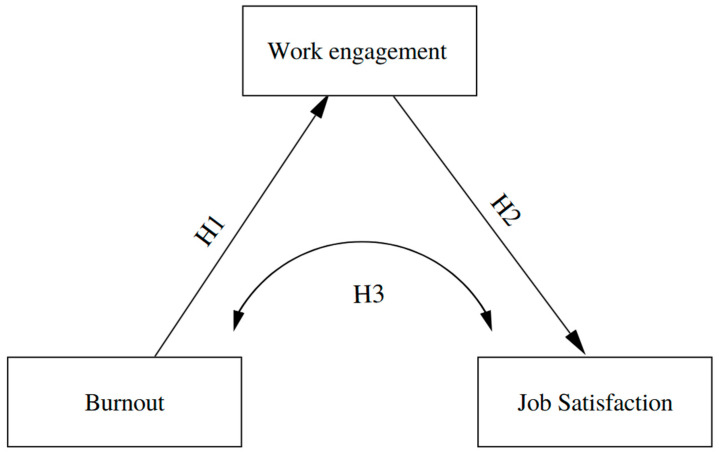
Theoretical model.

Both international and national literature allow us to anticipate that burnout is negatively related to work engagement, that engagement is positively associated with job satisfaction, and that, in line with the JD-R model, engagement may function as a key mediator in the relationship between burnout and satisfaction. Nevertheless, in the Peruvian context there are still few studies that explicitly test this full mediation model among nurses using structural equation modeling, which limits understanding of the psychosocial mechanisms that link job demands to affective and attitudinal outcomes in this professional group. Based on the foregoing, the following hypotheses are proposed (Figure 1):

**Hypothesis** **1.** *Burnout will have a negative effect on work engagement*.

**Hypothesis** **2.** *Work engagement will have a positive effect on job satisfaction*.

**Hypothesis** **3.** *Work engagement will mediate the relationship between burnout and job satisfaction*.

## 2. Methods

### 2.1. Design and Population

A cross-sectional and explanatory study was designed, employing a structural equation modeling (SEM) approach to represent latent variables [28]. The sample size was estimated using the statistical calculation software developed by Soper [29] for structural equation models, which indicated a minimum required sample of 119 participants. This estimate considered the number of observed and latent variables, an anticipated effect size (λ = 0.30), a statistical power level (1 − β = 0.80), and a desired significance level (α = 0.05). Participants were recruited through non-probabilistic convenience sampling from public and private hospitals located in Lima Metropolitana (Peru). To preserve the institutional anonymity requested by the participating centers, the specific names of the hospitals are not reported; however, all are secondary and tertiary care facilities that provide inpatient services. The final sample consisted of 230 Peruvian nurses, aged between 22 and 68 years (M = 41.22, SD = 11.65). The majority were women (93.0%). Regarding marital status, most participants reported being single (48.3%). In terms of educational level, the majority had university-level education (79.6%). Regarding employment status, the highest proportion were employed under the CAS contract scheme (55.2%) (see Table 1).

### 2.2. Measures

*Job Satisfaction:* Job satisfaction was assessed using the Spanish version of the G_Clinic questionnaire, originally developed and validated with nursing professionals working in clinical management units of the Andalusian public healthcare system [30]. The instrument consists of 10 items distributed across four dimensions: work climate, work relationships, motivation, and recognition. In its original study, it showed adequate global fit indices and acceptable internal consistency (overall α = 0.75; dimensions ≥ 0.70) [30]. The scale uses a 5-point Likert-type response format, where 1 indicates lower agreement or satisfaction and 5 indicates higher agreement or satisfaction, such that higher scores reflect higher levels of job satisfaction. In the present study, a confirmatory factor analysis yielded a satisfactory fit for the four-factor model: χ^2^(29) = 48.24, *p* = 0.014, CFI = 0.98, TLI = 0.97, RMSEA = 0.05 (90% CI [0.03, 0.07]), and SRMR = 0.03. The internal consistency of the dimensions was excellent, with Cronbach’s alpha (α) coefficients of 0.92, 0.92, 0.83, and 0.94, and omega (ω) coefficients of 0.92, 0.92, 0.84, and 0.94 for work climate, work relationships, motivation, and recognition, respectively, supporting the reliability of the scale in this sample of nurses.

*Work Engagement:* Work engagement was assessed using the 9-item short version of the Utrecht Work Engagement Scale (UWES-9), previously validated among healthcare professionals in Mexico [31] and adapted to the Peruvian work context [32]. This scale measures a positive affective–cognitive state of connection with one’s work, comprising three dimensions: Vigor, Dedication, and Absorption. Items are rated on a 6-point Likert-type scale ranging from 0 (never) to 5 (always), where higher scores indicate higher levels of work engagement. In the present study, the three-factor model of the UWES-9 showed an acceptable fit to the data: χ^2^(24) = 59.48, *p* < 0.001, CFI = 0.97, TLI = 0.96, RMSEA = 0.08 (90% CI [0.06, 0.10]), and SRMR = 0.02. The internal consistency of the three dimensions was excellent, with Cronbach’s alpha (α) coefficients of 0.94, 0.97, and 0.92, and omega (ω) coefficients of 0.94, 0.97, and 0.92 for Vigor, Dedication, and Absorption, respectively, which supports the reliability of the instrument in this sample of nurses.

*Burnout:* The Single-Item Burnout Scale (Ítem Único de Burnout, IUB) was validated in a sample of Peruvian workers [33]. This scale consists of a single item that assesses burnout globally, without subdivision into dimensions, and uses a 5-point ordinal Likert-type scale ranging from “not feeling burned out” to “feeling completely burned out.”

### 2.3. Procedure

Authorization was subsequently obtained from the administrations of two hospitals to conduct the study. Data collection took place between January and February 2025, with voluntary participation from nurses who completed the survey via Google Forms, allowing for online distribution. Prior to data collection, ethical guidelines established in the Declaration of Helsinki were followed, ensuring data confidentiality. Each participant was informed of the study’s nature and provided informed consent. Finally, the completeness of the submitted questionnaires was verified, which are presented below.

### 2.4. Data Analysis

The theoretical model was analyzed using structural equation modeling (SEM), employing the MLR estimator, which is suitable for continuous variables and robust to deviations from inferential normality [34]. Model fit was evaluated using the following indices: Comparative Fit Index (CFI), Root Mean Square Error of Approximation (RMSEA), and Standardized Root Mean Square Residual (SRMR). Acceptable thresholds were set as follows: CFI and TLI > 0.90 [35], RMSEA < 0.080 [36], and SRMR < 0.080 [37].

For mediation analysis, the bootstrapping method was applied with 5000 iterations and a 95% confidence interval [38]. Regarding the reliability of the scales, internal consistency was assessed using Cronbach’s alpha (α) and omega (ω) coefficients, following McDonald’s recommendations [39].

The SEM analysis was conducted using R software (version 4.0.5) with the *lavaan* package [40].

## 3. Results

### 3.1. Preliminary Analysis

Table 2 presents descriptive statistics and correlations among job satisfaction, work engagement, and burnout. Overall, the three variables exhibited approximately normal distributions, with skewness (g1) and kurtosis (g2) values within acceptable ranges. Job satisfaction and work engagement were positively correlated (r = 0.79, *p* < 0.01). Conversely, burnout showed negative correlations with both job satisfaction (r = −0.41, *p* < 0.01) and work engagement (r = −0.47, *p* < 0.01).

### 3.2. Structural Model

**Figure 2:**
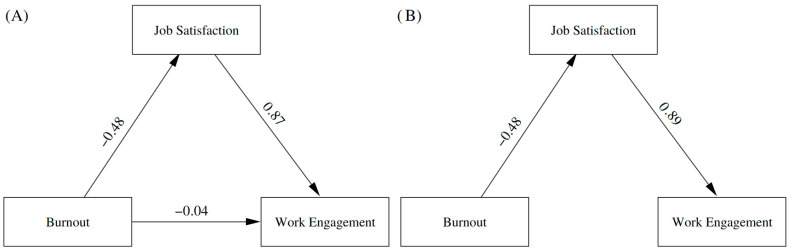
Theoretical structural models. (**A**) Structural model including the direct and indirect effects of burnout on job satisfaction through work engage-ment (partial mediation model). (**B**) Structural model with the direct path from burnout to job satisfaction constrained to zero, representing the full mediation model through work engagement.

In the theoretical analysis of the model, an adequate fit was obtained: χ^2^ = 284.820, df = 161, *p* = 0.000; CFI = 0.96, TLI = 0.95, RMSEA = 0.06 (90% CI: 0.05–0.07), SRMR = 0.04. These results are reflected in the model on the left side of Figure 2 (Model A), where a significant indirect effect of burnout on job satisfaction through work engagement is observed. However, the direct effect of burnout on job satisfaction was very small (β = −0.04), suggesting that the relationship is fully mediated by work engagement. For reasons of parsimony, this direct effect was constrained to zero in Model B (right side of the figure), which also showed a good fit with identical fit indices (χ^2^ = 284.820, df = 161, *p* = 0.000; CFI = 0.96, TLI = 0.95, RMSEA = 0.06 [90% CI: 0.05–0.07], SRMR = 0.04). These findings support the hypothesis of full mediation, in which work engagement entirely explains the relationship between burnout and job satisfaction.

### 3.3. Mediation Analysis

Bootstrapping with 5000 resamples was used for the mediation analysis. Regarding H3, the mediating effect of work engagement in the relationship between burnout and job satisfaction was confirmed, with a significant indirect effect (β = 0.43, 95% CI [0.21, 0.44], *p* < 0.001). The model explained 23.7% of the variance in work engagement (R^2^ = 0.24) and 79.6% of the variance in job satisfaction (R^2^ = 0.80). Standardized effects are presented in Table 3.

## 4. Discussion

The findings of this study confirmed Hypothesis 1, showing that higher levels of burnout are associated with lower work engagement among nurses, weakening their affective and cognitive connection to their work. This aligns with the Job Demands–Resources (JD-R) Model, which posits that excessive demands without sufficient resources erode engagement. This result is consistent with international evidence documenting that high emotional demands, workload, and care pressure increase burnout and reduce engagement, which in turn heightens turnover intention and the likelihood of leaving the profession [4,41]. In the Latin American context, the literature shows that nurses’ quality of work life often falls within medium or low ranges, with clear repercussions for their health and the quality of care [42]. In Peru, burnout has been linked to the intention of healthcare professionals to migrate, aggravating staffing shortages and destabilizing care teams [43]. Our results expand this landscape by demonstrating, through a structural model, that burnout not only impacts individual distress but also directly undermines work engagement—a key resource for sustaining performance and retention in care services. From a hospital management perspective, this implies that strategies to contain burnout (e.g., regulating workloads, adequate nurse-to-patient ratios, effective rest periods, and psychosocial support) are not merely well-being interventions, but structural actions to preserve engagement and, consequently, reduce turnover and the risk of losing specialized talent.

Regarding Hypothesis 2, the results showed that work engagement has a positive and significant effect on job satisfaction, confirming that nurses who feel vigorous, dedicated, and absorbed in their work report higher levels of job satisfaction. This finding reinforces the central premise of the JD-R model: job resources (autonomy, leadership support, positive climate, development opportunities) foster engagement and, through it, increase satisfaction and psychological well-being. Recent studies have shown that when nurses perceive a favorable work environment, including manageable workloads, clear task delegation, and less disruptive shifts, their satisfaction increases and their intention to leave decreases [44]. In the Peruvian context, job satisfaction among healthcare professionals has been strongly shaped by overload, contractual instability, and heightened pressure during and after the pandemic [45], and low satisfaction levels have been linked to greater intention to resign among Peruvian nurses [46]. The present study provides additional evidence by showing that even in a high-demand setting with limited resources, work engagement can function as a positive mechanism that channels organizational resources into greater satisfaction. This has direct implications for staff retention, continuity of care, and service quality. For hospital managers, this suggests that investing in transformational leadership practices, explicit recognition, training opportunities, and participation in decision-making not only improves the work climate but also translates into increased engagement and, ultimately, higher satisfaction and lower turnover intention.

Finally, Hypothesis 3 was confirmed by the finding that work engagement significantly mediates the relationship between burnout and job satisfaction, resulting in a full mediation model in which the effect of burnout on satisfaction is channeled through engagement. In practical terms, this means that burnout tends to reduce job satisfaction, but its impact can be mitigated when nurses maintain high levels of engagement—positioning engagement as a strategic leverage point for hospital management. This result aligns with recent meta-analyses showing that nurse burnout is associated with lower quality of care, more adverse events, and reduced patient satisfaction [47], as well as with evidence that healthier and more organized work environments reduce burnout and improve satisfaction and retention [44,48]. In the Latin American context, where medium-to-low levels of quality of work life and their impact on staff health have been documented [42], our findings add a relevant nuance: among Peruvian nurses, work engagement is not only associated with greater satisfaction but also acts as a buffer against strain, offering an explanatory mechanism for why some teams maintain acceptable satisfaction levels even under adverse conditions. From a policy and management standpoint, this underscores the need for comprehensive interventions that combine reducing job demands (e.g., excessive shifts, mandatory overtime, insufficient staffing) with strengthening resources that promote engagement (inspirational leadership, social support, well-being programs, recognition, and professional development). Such strategies could not only reduce burnout and improve satisfaction but also decrease turnover intention and nurse migration, contributing to workforce stability and to the quality and safety of care in Peruvian hospitals.

### 4.1. Implications

The findings of this study provide robust empirical evidence on the relationship between burnout, work engagement, and job satisfaction among nursing personnel, with direct implications for clinical practice, hospital management, and the design of organizational policies. First, the results support the need to design and implement training and development programs based on work engagement and aligned with the JD-R model, aimed at strengthening resources such as vigor, dedication, and absorption. These programs may include workshops on stress regulation, active coping strategies, communication skills, and the construction of meaning in work, integrated into the continuous professional development of nursing staff. Second, from a hospital management perspective, it is recommended to incorporate psychosocial well-being indicators (burnout, engagement, and job satisfaction) into institutional evaluation systems and human resources dashboards so that these indicators are periodically monitored alongside classic parameters such as turnover, absenteeism, adverse events, and quality of care. This would help identify units or services at risk and prioritize interventions aimed at reducing overload and improving working conditions (e.g., staffing adjustments, shift reorganization, access to psychological support). Third, the results suggest that organizational policies should emphasize strengthening key job resources: transformational and supportive leadership styles, structural empowerment (participation in decision-making, autonomy in practice), and explicit recognition of performance. Concrete actions include leadership training programs for nursing supervisors, recognition systems that are not only financial but also symbolic, and clear pathways for professional development. By increasing resources, work engagement is strengthened, which—according to the JD-R model and the findings of this study—can buffer the impact of burnout and foster satisfaction and staff retention. Finally, at the theoretical level, these results expand the evidence on the Job Demands–Resources Model in a Latin American context characterized by high care demands, showing that work engagement functions as a key mediator between burnout and job satisfaction. This reinforces the importance of considering engagement not only as a desirable outcome but also as a central mechanism through which management policies and practices can translate into well-being and sustainability in nursing work.

### 4.2. Limitations

This study has several limitations that should be considered when interpreting the results. First, the cross-sectional design prevents establishing causal relationships between burnout, engagement, and job satisfaction; therefore, the observed associations should be interpreted as correlational. Longitudinal or experimental studies would allow examination of the directionality of effects and the evolution of these variables over time. Second, a non-probabilistic sample of nurses from specific institutions was used, which limits the generalizability of the findings to the entire population of Peruvian nurses. It is possible that participants differed from non-respondents in relevant variables (e.g., motivation, availability of time, or satisfaction level), introducing potential selection bias. Future studies should consider probabilistic sampling strategies and larger multicenter samples. Third, all variables were measured through self-report, which may generate social desirability and common method biases, given that the information source is the same and responses may be influenced by mood or perceptions of the institution. Incorporating objective indicators (e.g., turnover and absenteeism rates, patient complaints) and hetero-reported evaluations (e.g., supervisor-rated performance) would help increase the validity of the results. Fourth, although the use of a unidimensional burnout measure based on a single item has prior evidence of acceptable validity and reliability for capturing the global experience of feeling “burned out,” it represents an important limitation in this study. This measure restricts analysis of the classical dimensions of the construct (emotional exhaustion, depersonalization, and reduced personal accomplishment) and may underestimate the complexity of the phenomenon, as well as limit the variance available for structural modeling. Future studies should employ multidimensional burnout scales (for example, abbreviated MBI versions or other instruments validated in the Peruvian context), which would allow for comparison with our findings, analysis of differentiated burnout profiles, and more precise exploration of its relationship with engagement and job satisfaction. Finally, reporting bias is possible (e.g., underreporting burnout symptoms due to fear of stigma or workplace repercussions), as is a potential healthy worker effect, given that those with the most severe levels of strain may have been absent or not have participated in the study. These limitations suggest interpreting the results with caution and reinforce the need to replicate the model in different contexts and with complementary methodologies.

## 5. Conclusions

The findings of this study provide solid empirical evidence on the relational dynamics between burnout, work engagement, and job satisfaction among nursing personnel, confirming a full mediation model in which work engagement acts as a protective mechanism against the negative impact of burnout on job satisfaction. This evidence strengthens the theoretical framework of the Job Demands–Resources (JD-R) Model and highlights the central role of engagement as a key psychosocial resource for well-being in highly demanding care settings. In practical terms, the results support the implementation of concrete organizational interventions that strengthen work engagement, such as (a) training and development programs based on engagement and aligned with the JD-R model, integrating demand management, resource strengthening, and transformational leadership development; (b) systematic incorporation of well-being indicators (burnout, engagement, and satisfaction) into institutional evaluations and human resource monitoring systems; and (c) structural empowerment and recognition policies that promote self-efficacy, participation in decision-making, and a sense of purpose at work. Taken together, these strategies may help not only reduce burnout and improve job satisfaction but also decrease turnover intention, stabilize nursing staffing, and ultimately enhance the quality and safety of care in Peruvian hospitals.

## Figures and Tables

**Table 1:** Sociodemographic Characteristics.

Characteristics		*n*	%
Sex	Female	214	93.0
	Male	16	7.0
Marital Status	Married	62	27.0
	Cohabiting	47	20.4
	Divorced	8	3.5
	Single	111	48.3
	Widowed	2	0.9
Educational Level	Professional Specialization	27	11.7
	Graduate Studies	20	8.7
	University Degree	183	79.6
Employment Status	Permanent Contract 728	19	8.3
	CAS Contract	127	55.2
	Tenured	59	25.7
	Substitute	2	0.9
	Outsourced	23	10.0

**Table 2:** Descriptive statistics and correlations.

Variables	M	SD	g1	g2	1	2	3
1. Job satisfaction	24.74	9.67	0.44	−0.85	**−**		
2. Work engagement	32.64	10.38	0.36	−0.72	0.79 **	**−**	
3. Burnout	1.85	0.96	0.09	−0.2	−0.41 **	−0.47 **	**−**

Note: ** indicates *p* < 0.01, M = Mean, SD = Standard Deviation, g1 = Skewness, g2 = Kurtosis.

**Table 3:** Mediation model.

Relationship	Type of Effect	Standardized β	95% CI Lower	95% CI Upper	*p*
Burnout → Work engagement	Direct	0.486	0.401	0.843	<0.001
Work engagement → Job satisfaction	Direct	0.892	0.43	0.615	<0.001
Burnout → Job satisfaction (via engagement)	Indirect ^1^	0.434	0.214	0.444	<0.001
Burnout → Job satisfaction	Total ^2^	0.434	0.214	0.444	<0.001

Notes. ^1^ Indirect effect (a × b) of burnout on job satisfaction mediated by work engagement. ^2^ In this full mediation model, no direct effect of burnout on job satisfaction was estimated; therefore, the total effect matches the indirect effect.

## Data Availability

The datasets generated and analyzed during the current study are not publicly available due to ethical and confidentiality restrictions but are available from the corresponding author on reasonable request.

## Public Involvement Statement

No public involvement in any aspect of this research.

## Guidelines and Standards Statement

This manuscript was drafted in accordance with the STROBE (Strengthening the Reporting of Observational Studies in Epidemiology) guidelines for observational research.
